# Calcium Sets the Clock in Ameloblasts

**DOI:** 10.3389/fphys.2020.00920

**Published:** 2020-07-31

**Authors:** Raed Said, Liubov Lobanova, Silvana Papagerakis, Petros Papagerakis

**Affiliations:** ^1^Department of Anatomy, Physiology and Pharmacology, College of Medicine, University of Saskatchewan, Saskatoon, SK, Canada; ^2^College of Dentistry, University of Saskatchewan, Saskatoon, SK, Canada; ^3^Department of Surgery, College of Medicine, University of Saskatchewan, Saskatoon, SK, Canada

**Keywords:** ameloblast, enamel, STIM1, calcium, store operated Ca^2+^ channels, circadian clock, amelogenesis, amelogenesis imperfecta

## Abstract

**Background:**

Stromal interaction molecule 1 (*STIM1*) is one of the main components of the store operated Ca^2+^ entry (SOCE) signaling pathway. Individuals with mutated *STIM1* present severely hypomineralized enamel characterized as amelogenesis imperfecta (AI) but the downstream molecular mechanisms involved remain unclear. Circadian clock signaling plays a key role in regulating the enamel thickness and mineralization, but the effects of *STIM1*-mediated AI on circadian clock are unknown.

**Objectives:**

The aim of this study is to examine the potential links between SOCE and the circadian clock during amelogenesis.

**Methods:**

We have generated mice with ameloblast-specific deletion of *Stim1* (*Stim1*^fl/fl^/Amelx-iCre^+/+^, *Stim1* cKO) and analyzed circadian gene expression profile in *Stim1 cKO* compared to control (*Stim1*^fl/fl^/Amelx-iCre^–/–^) using ameloblast micro-dissection and RNA micro-array of 84 circadian genes. Expression level changes were validated by qRT-PCR and immunohistochemistry.

**Results:**

*Stim1* deletion has resulted in significant upregulation of the core circadian activator gene Brain and Muscle Aryl Hydrocarbon Receptor Nuclear Translocation 1 (*Bmal1*) and downregulation of the circadian inhibitor Period 2 (*Per2*). Our analyses also revealed that SOCE disruption results in dysregulation of two additional circadian regulators; p38α mitogen-activated protein kinase (MAPK14) and transforming growth factor-beta1 (TGF-β1). Both MAPK14 and TGF-β1 pathways are known to play major roles in enamel secretion and their dysregulation has been previously implicated in the development of AI phenotype.

**Conclusion:**

These data indicate that disruption of SOCE significantly affects the ameloblasts molecular circadian clock, suggesting that alteration of the circadian clock may be partly involved in the development of *STIM1*-mediated AI.

## Introduction

Calcium plays a pivotal role in enamel mineralization, yet the exact mechanisms involved in Ca^2+^ transport and the roles of Ca^2+^ signaling in regulating ameloblast functions during amelogenesis remain unclear (Berdal et al., 1995; Bailleul-Forestier et al., 1996; Nurbaeva et al., 2017). Initially, Ca^2+^ influx into ameloblasts was thought to occur passively via paracellular diffusion (Bawden, 1989). However, several subsequent reports have shown that Ca^2+^ transport occurs principally transcellularly and mainly through high capacity intracellular stores in endoplasmic reticulum (ER) (Hubbard, 2000; Lacruz et al., 2012b; Nurbaeva et al., 2015a, b, 2017). This mode of transport is termed Store-operated Ca^2+^ entry (SOCE) and is mainly mediated by the ER transmembrane proteins Stromal interaction molecule 1 and 2 (STIM1 and STIM2) and the highly selective plasma membrane (PM) calcium release activated channels (CRAC) (Prakriya and Lewis, 2015; Lacruz, 2017). STIM1 and STIM2 serve as intracellular (i) [Ca^2+^] sensors, while CRAC protein 1, 2 and 3 (ORAI1, 2, and 3) are transmembrane proteins that form the pores of the CRAC channels and serve as a highly selective filter during Ca^2+^ entry from the circulation to the ameloblasts (Lacruz, 2017; Fahrner et al., 2018). Upon Ca^2+^ depletion from the ER, STIM1 binds to ORAI to facilitate Ca^2+^ entry and activate Ca^2+^ -dependent signal transduction. It must be noted that STIM2 is usually present at lower levels than STIM1, and that ORAI2 and ORAI3 activation results in a lower Ca^2+^ intake compared with ORAI1 (Hoth and Niemeyer, 2013). The importance of SOCE in regulating calcium transport during enamel mineralization is clearly reflected in the fact that patients with mutations in either STIM1 or ORAI1 have severe dental enamel defects, characterized as hypocalcified Amelogenesis Imperfecta (AI) (Eckstein and Lacruz, 2018; Feske, 2019). Moreover, several *in vivo* and *in vitro* studies have shown that SOCE components are robustly expressed by ameloblasts and served as key modulators of ameloblast’s differentiation and function (Nurbaeva et al., 2015a, b; Zheng et al., 2015; Eckstein et al., 2017; Furukawa et al., 2017; Said et al., 2019).

It is well established that Ca^2+^ may act as a second messenger with a broad role in regulating gene expression *via* orchestrating several major signaling pathways (Tonelli et al., 2012). One of the main molecular systems essential for amelogenesis is the circadian clock (Athanassiou-Papaefthymiou et al., 2011). Circadian clock is an intracellular mechanism that regulates gene and protein expression levels oscillations over a 24-h period (Adeola et al., 2019). These circadian genetic rhythms are directly controlled by a complex system of body clocks, which entails a central or master clock located in the hypothalamic suprachiasmatic nucleus (SCN) and several subordinate peripheral clocks located in multiple tissues including in ameloblasts (Adeola et al., 2019). At the molecular level, circadian rhythms are maintained *via* the differential expression of several transcription factors called clock genes (Partch et al., 2014; Takahashi, 2017). The main mammalian clock genes include Circadian Locomotor Output Cycles Kaput (Clock), Brain and Muscle Aryl Hydrocarbon Receptor Nuclear Translocation (Arntl or Bmal1), Period 1 (Per1), Period 2 (Per2), Period 3 (Per3), and Cryptochromes (Cry1) and Cry2 (Partch et al., 2014). Clock genes interact with each other in an intricate manner and form perpetual autoregulatory transcription translation feedback loops (TTFLs) that control the rhythmic expression of several clock-controlled genes (CCG) to create 24-h repetitive expression patterns necessary for normal functions in several physiological processes (Takahashi, 2017). TTFLs are affected directly by extracellular events whose actions are transduced intracellularly by second messengers such as calcium and cAMP (Honma and Honma, 2003; Hastings et al., 2018). In mammalian tissues, several studies showed that calcium can act directly on TTFLs via the calcium/cAMP-dependent transcription factor (CREB) (O’Neill and Reddy, 2012; Herzog et al., 2017). In the mouse SCN, it was shown that blocking calcium influx and lowering i[Ca^2+^] had abolished the rhythmic expression of *Per1* (Lundkvist et al., 2005). In addition, the amplitude of *Per1* and *Per2* expression was significantly decreased by voltage-gated Ca^2+^ channel antagonists (Lundkvist et al., 2005). On the other hand, it must be noted that i[Ca^2+^] and cAMP levels oscillate in a circadian manner in the SCN neurons (O’Neill and Reddy, 2012). Thus, it has been suggested that cAMP and Ca^2+^ signaling may not only contribute in regulating timekeeping, but also that they are regulated by the cellular clock (i.e., Ca^2+^ and cAMP signaling is both output from, as well as input into the core clock pathway) (Palacios-Muñoz and Ewer, 2018).

The periodical nature of enamel secretion and the synchronized sequential pattern of ameloblast differentiation and mineralization strongly suggest that the process of amelogenesis is under circadian regulation (Papagerakis et al., 2014). Moreover, several publications from our group and others showed that this time-related control of ameloblasts activities may be orchestrated by the differential expression of circadian clock proteins during the distinct stages of enamel formation (Simmer et al., 2010; Athanassiou-Papaefthymiou et al., 2011; Lacruz et al., 2012a; Zheng et al., 2013, 2014). However, the molecular mechanisms involved in regulating the circadian clock during amelogenesis remain largely unexplored. In this study, we aimed to investigate the effects of calcium disruption on the ameloblasts molecular clock *in vivo* using a unique *Stim1*^fl/fl^/Amelx-iCre^+/+^ (*Stim1* cKO) model (Said et al., 2019). The significant impact of Ca^2+^ and circadian clock in ameloblasts makes amelogenesis an excellent model system for deciphering the mechanistic links that may exist between intracellular calcium dynamics and molecular circadian clock. Indeed, our analysis showed that *Stim1* ablation in ameloblasts results in significant dysregulation of the ameloblasts’ circadian clock. More specifically, we found that loss of *Stim1* results in significant upregulation of the master circadian clock gene *Bmal1* and down regulation of its antagonist, *Per2*. We also found that targeting *Stim1* leads to significant changes of several other circadian regulators, including the p38α mitogen-activated protein kinase (MAPK14) and transforming growth factor-beta1 (TGF-β1). Both MAPK14 and TGF-β1 pathways are known to play a key role in tooth morphogenesis and enamel secretion, and their dysregulation has been previously implicated in the development of AI phenotype. Our data strongly suggest that SOCE affects the molecular circadian clock in ameloblast, which could be one of the downstream pathways involved in the development of *Stim1*-mediated AI.

## Materials and Methods

### Animals

*Stim1* cKO generation and characterization were previously described (Said et al., 2019). Animals were housed in a pathogen-free facility in the Lab Animal Service Unit (LASU) at the University of Saskatchewan and all procedures for this study were authorized by the University Animal Care Committee (UACC) under an approved protocol (#20170014). All mice were housed under standard laboratory conditions in light-dark (LD) 12:12 conditions, normal room temperature, with *ad libitum* access to food and water. Genotypes were determined by tail biopsy and conventional polymerase chain reaction (PCR). The mice were humanely culled at postnatal day 14 (P14).

### PCR Arrays

Enamel organ and secretory ameloblasts were micro-dissected from 2-week-old incisors in the left hemimandibles as previously described (Houari et al., 2018) and used for RNA extraction. RNA from *Stim1* cKO and control animals was extracted using the RNeasy MiniKit (Qiagen). cDNA was made using RT2 First Strand Kit (Qiagen). The cDNA was used on the real-time RT^2^ Profiler PCR Array (QIAGEN, Cat. no. PAMM-153Z) in combination with RT^2^ SYBR Green qPCR Mastermix (Cat. no. 330529). CT values were exported to an Excel file to create a table of CT values. This table was then uploaded onto the data analysis web portal at http://www.qiagen.com/genelobe. Samples were assigned to controls and test groups. CT values were normalized based on a manual selection of two reference genes (HKG), B-actin (*Actb*) and Glyceraldehyde 3-phosphate dehydrogenase (*Gapdh*). The CT cut-off was set to 35. The data analysis web portal calculated fold change/regulation using ΔΔCT method, in which ΔCT is calculated between gene of interest (GOI) and an average of reference genes (HKG), followed by delta-delta CT (ΔΔCT) calculations [ΔCT (Test Group)– ΔCT (Control Group)]. Fold Change was then calculated using 2^ (−ΔΔCT) formula. The data analysis web portal also plots scatter plot, volcano plot, clustergram, and heat map. DAVID software tool was used to analyze the examined genes (Huang et al., 2009).

### qRT-PCR

RNA was extracted with the RNeasy MiniKit (Qiagen) from *Stim1* cKO and control mice (*Stim1*^fl/fl/^Amelx-iCre^–/–^) (*n* = 5 per genotype). After cDNA synthesis, the gene expression of *Bmal1*, *Clock*, *Per1 & 2*, *Cry 1&2*, *Mapk14*, *Tgf-*β*1*, Signal Transducer, and Activator of Transcription 5A (*Stat5a*), F-Box and Leucine Rich Repeat Protein (*Fbxl*) 3, RAR-related orphan receptor alpha (*Rora*), Nuclear Receptor Subfamily 1 Group D Member 1 (*NR1D1*), was assessed by quantitative PCR (qRT-PCR) with the 2^(−ΔΔCt) method. *Gapdh* was used as the reference gene. All primer sequences are included (Supplementary Table 1).

### Statistics

All data are presented as mean ± standard deviation (SD). Student’s *t*-test was used to compare the gene expression. For the PCR profiler assays, fold-change values greater than 2 or less than 0.5 were considered differentially expressed. The *P*-values were calculated based on a Student’s *t*-test of the replicate 2^(−ΔΔCt) values for each gene in the control and *Stim1* cKO groups; *P*-value < 0.05 was considered significant.

### Immunohistochemistry

The right hemimandibles were sectioned in a sagittal plane and immunostained to assess BMAL1, PER2, and *STIM1* protein cellular and tissue localization with a rabbit anti-PER2 antibody (1:200, LS-C2836; LifeSpan Biosciences), a rabbit anti-BMAL1 (1:250, NB100-2288; Novus Biologicals) and a rabbit anti-STIM1 (1:200, LSC34692; LSBio).

## Results

### The Circadian Profile of Ameloblasts Changes Significantly After *Stim1* Deletion

**FIGURE 1:**
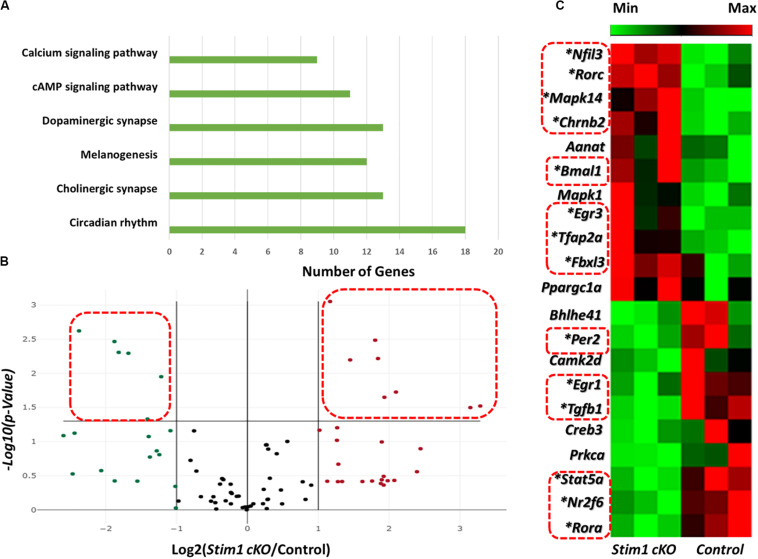
Effects of *Stim1* KO on the gene expression profiles in ameloblasts. The expression change of 84 genes in ameloblasts from *Stim1* cKO and control mice was performed by real-time PCR based array analysis. **(A)** A bar diagram showing the functional clustering of the examined genes in the 6 most enriched signal transduction pathways according to the Gene Ontology and KEGG pathway analysis performed by the DAVID software **(B)** A volcano plot for the examined genes in total, black dots indicates genes with unchanged gene expression in Stim 1 cKO versus control; the genes with at least two fold change [(i.e., Log2 (*Stim1 cKO*/Control) > 1 or < −1] were represented as red dots for the upregulated genes and in green dots for the downregulated genes; the genes were deemed to be significantly differentially expressed if presented with at least two fold change and a *P-*value < 0.05 [i.e., −Log_10_(*P*-Value) > 1.305, represented by the dots within the dashed red rectangles]. **(C)** A cluster heatmap showing the expression changes in some of the examined genes in *Stim1* cKO ameloblasts against the control group. A total of 14 genes were found to be significantly differentially expressed in *Stim1* cKO ameloblasts compared to the control group (*P* < 0.05, labeled by asterisks and within dashed red rectangles).

We first assessed the effect of *Stim1* ablation on ameloblasts’ circadian clock signaling by performing a validated circadian pathway PCR RNA microarray analysis. This analysis enabled us to evaluate the mRNA levels of 84 different genes in the circadian network. Gene Ontology and KEGG pathway analysis in the DAVID software (Huang et al., 2009) showed that the examined genes function in several essential signaling pathways including many calcium dependent ones (Figure 1A). Compared to control, we found that fourteen circadian genes were significantly differentially expressed (*P* < 0.05) in *Stim1* cKO ameloblasts with at least a twofold differential expression compared to control (Figures 1B,C, Supplementary Table 2, and Supplementary Datasheet 1). Specifically, we found that *Stim1* disruption had led to significant changes in the expression levels of several key clock genes that form the circadian loops, including *Bmal1*, *Per2*, *Fbxl3*, *Ror-a*, *and Ror-c*, which strongly indicates that SOCE may influence the ameloblast circadian clock system. The most significant changes were observed in *Bmal1 and Per2* expression, as *Bmal1* expression was greatly upregulated while *Per2* was greatly downregulated in *Stim1* cKO ameloblasts. We also found that *Stim1* knock out resulted in significant changes in the expression levels of circadian regulators that are also known to be involved in regulating amelogenesis throughout its different stages whether it is during early craniofacial development (Transcription Factor AP-2 Alpha, *Tfap2a*), tooth morphogenesis and early enamel secretion (*Mapk14*), or enamel maturation and mineralization (*Tgf-* β1). Additionally, the RNA profile revealed that SOCE disruption altered the expression of other circadian transcription factors including, the nuclear receptor subfamily 2 group F member 6 (NR2F6) and early growth response proteins 1 & 3 (EGR1, EGR3).

### Validation of *Stim1* Deletion Effects on the Ameloblasts Circadian Clock

**FIGURE 2:**
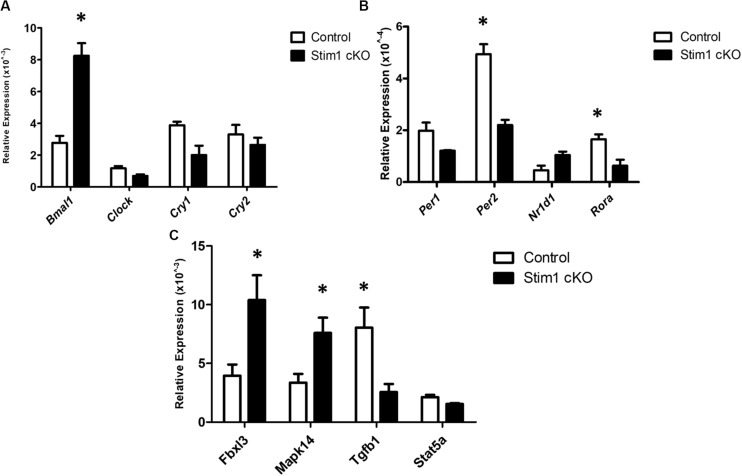
The validation for selected candidate genes by qRT-PCR **(A–C)**. Data showed the relative mRNA fold changes of candidate genes in *Stim1* cKO groups versus the control groups (*n* = 5 per genotype). All experiments were performed in triplicates (^∗^*P* < 0.05).

We further validated these substantial changes in the circadian clock gene expression by qRT-PCR analysis in which we first assessed the differences in the mRNA levels of 8 core clock genes (Figures 2A,B). Similar to profiler RNA micro-array analysis, the qRT-PCR analysis showed that *Bmal1* was greatly upregulated in *Stim1* cKO mice while both *Per2* and *Ror-a* were significantly downregulated. In addition to examining the core circadian genes we further assessed the changes of four additional circadian clock related genes of interest (Figure 2C). Similar to the profiler RNA micro-array pathway analysis, the qRT-PCR analysis showed that loss of *Stim1* function in ameloblasts significantly alters the expression pattern of *Tgf-*β*1* (downregulated) and *Mapk14* (upregulated) and *Fbxl3* (upregulated). It must be noted however, that contrary to the profiler assay analysis results, no significant changes were detected in the *Stat5a* gene.

### Examining the Clock Proteins in *Stim1* cKO Ameloblasts

**FIGURE 3:**
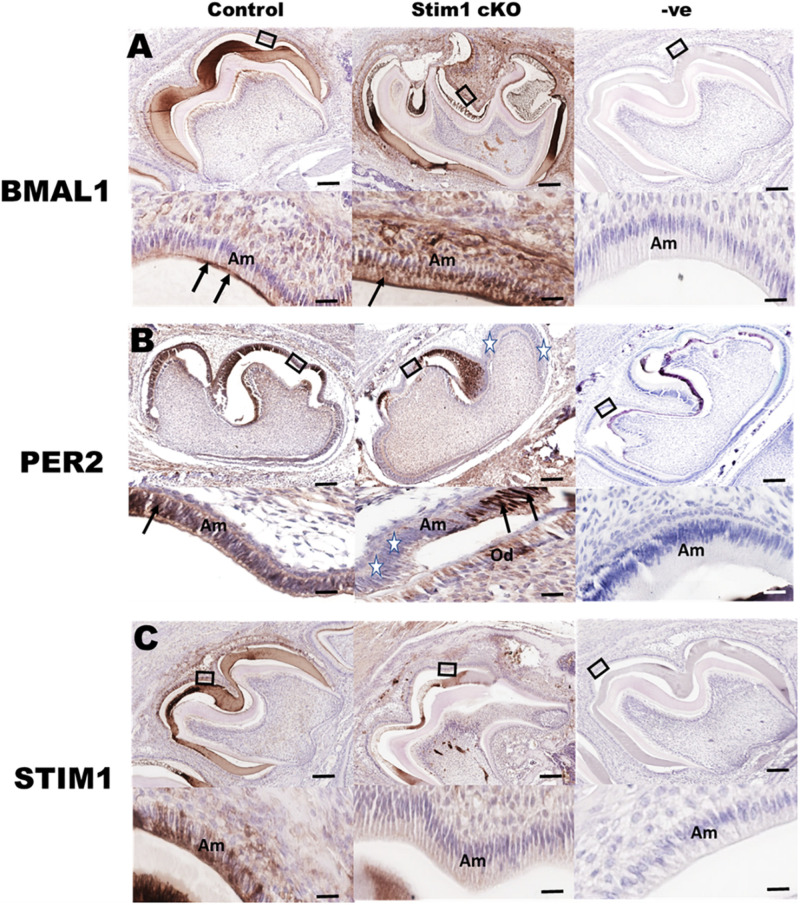
Immunohistochemical validation of gene expression data. Representative images of mouse molar sections stained using specific antibodies against BMAL1 **(A)**, PER2 **(B)**, and STIM1 **(C)**. BMAL1 proteins levels are higher in the *Stim1* cKO group compared to control and is mainly localized in the apical cytoplasm (arrows) of the ameloblasts. PER2 expression is weaker in the *Stim1* cKO compared to control and is localized in the nucleus (arrows). Very weak STIM1 signal can be detected in *Stim1* cKO ameloblasts which confirms the validity of our experimental model. The rectangular areas are enlarged in the lower panel of each image. White stars label the cells expressing very low levels of *Per2*. Am, ameloblasts; Od, odontoblasts. Scale bars, 250 μm in upper panels, 50 μm in lower panels.

Immunohistochemical analysis using specific antibodies against BMAL1, PER2, and STIM1 was performed in order to further validate the RNA data reported above and to confirm *Stim1* deletion in ameloblasts. Our data clearly showed that in *Stim1* cKO ameloblasts BMAL1 proteins levels were much higher compared to control (Figure 3A). The PER2 expression levels were clearly downregulated overall, although PER2 expression pattern was quite peculiar as it appears to be expressed strongly in some ameloblasts nuclei and very weakly in others (Figure 3B, stars). This heterogeneity of PER2 localization in *Stim1* cKO ameloblasts may be attributed to posttranslational modifications including abnormal protein sequestering and degradation. Finally, our data here clearly showed that PER2 protein is mainly located in the nucleus (Figure 3B arrows) while BMAL1 is concentrated in the apical cytoplasm (Figure 3A, arrows). We previously reported the alternating pattern of these BMAL1 and PER2 localizations, where BMAL1 is cytoplasmic in the morning (8:00 AM) and translocates into the nucleus at night (8:00 PM) (Zheng et al., 2014). All mice for the current study were euthanized at 1:00 PM which may explain the differential localization between PER2 and BMAL1 in our data. Finally, only a very weak STIM1 signal can be detected in *Stim1* cKO ameloblasts, which confirms the validity of our experimental model (Figure 3C).

## Discussion

In this study we investigated the possible links between two important molecular regulators of amelogenesis, SOCE and the circadian clock. Using the *Stim1* cKO model, we demonstrated that enamel specific disruption of SOCE had profound effects on the ameloblasts’ circadian gene profile as *Stim1*-deficient ameloblasts showed an altered expression of several key clock genes including *Bmal1*, *Per2*, *Ror-a*, and *Fbxl3*. Furthermore, our analyses demonstrated that SOCE disruption affected the expression levels of other circadian regulators that are known to be involved in regulating tooth morphogenesis and ameloblasts differentiation, such as p38 MAPK and TGF- β1. Collectively, these data strongly implicate SOCE as a vital modulator of the ameloblasts’ circadian clock. To the best of our knowledge, this is the first study to show evidence of a direct interplay between circadian clock and calcium signaling in ameloblasts.

Our PCR RNA array, qRT-PCR, and IHC analyses of core clock genes revealed that *Stim1* knockdown had significantly affected the expression of the core circadian genes *Bmal1* (upregulated) and *Per2* (downregulated). Both genes serve as essential components of the autoregulatory mammalian clock gene network. BMAL1 protein heterodimerizes in the cytoplasm with the other circadian activator CLOCK (or NPAS2 in neurons) and then translocate into the nucleus where they enhance the expression of their repressors PER 1&2 and CRY 1&2 *via* binding to an E-box sequence in their promoter region (Ye et al., 2014). Subsequently, PER and CRY proteins multimerize with another core circadian regulator, casein kinase I δ in the cytoplasm and enter the nucleus to inhibit the activity of BMAL1/CLOCK complexes (St. John et al., 2014). Both genes are involved in the circadian regulation of several physiological (e.g., bone metabolism) and pathological processes (e.g., development of cancer) (Adeola et al., 2019; Janjiæ and Agis, 2019). In enamel, we have previously demonstrated that both *Bmal1* and *Per2* are robustly expressed by pre-ameloblast and differentiating ameloblasts in both human and mice embryonic teeth in addition to mice postnatal teeth (Zheng et al., 2011, 2014). We and others have also shown that the expression levels and localization of their protein products in ameloblasts regularly alternates in a circadian manner (Lacruz et al., 2012a; Zheng et al., 2013). Preliminary *in vivo* and *vitro* analysis from our lab revealed that both genes may play a major role in regulating the expression of key enamel matrix proteins such as amelogenin, and proteases such as Kallikrein 4 (Klk4). Moreover, our preliminary *in vivo* analysis of *Per2* KO mice teeth showed abnormal enamel matrix formation associated with defective hypomineralized AI phenotype similar to AI observed in our *Stim1* cKO (unpublished data). All of the above-mentioned observations indicate that STIM1-caused dysregulation of the circadian clock network may lead to impaired enamel formation. Thus, the SOCE-mediated circadian disruption may be critical in the development of AI observed in patients with *STIM1/ORAI1* loss of function mutations.

**FIGURE 4:**
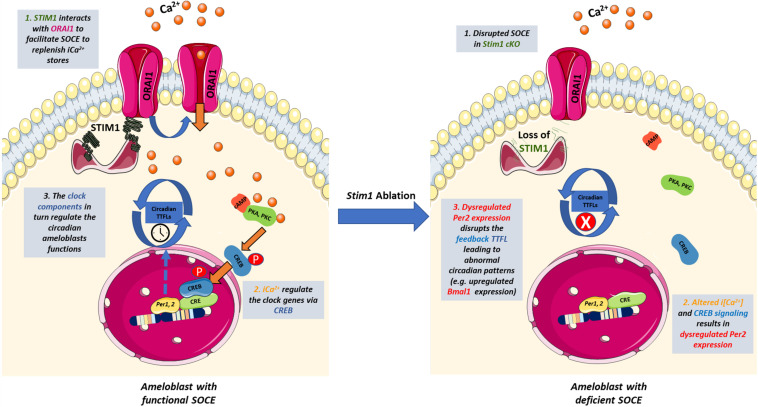
The potential cross-talk between SOCE and the circadian clock in ameloblasts. Schematic representation summarizing how SOCE can potentially influence the ameloblasts’ circadian clock based on our findings. Upon Ca^2+^ depletion in the ER, STIM1 binds to ORAI to facilitate Ca^2+^ entry and activate Ca^2+^-dependent signal transduction. Calcium induces the activity of Ca^2+^/calmodulin-dependent protein kinases which activate PKA and PKC that phosphorylate CREB in a circadian manner. The phosphorylated CREB translocate to the nucleus where it recognizes the cAMP Response Element in the promoter region of *Per1* & *2* and serves as a transcription factor. PER1 & 2 are core regulators of the mammalian circadian clock and participate in forming the autoregulatory TTFL. Disruption of SOCE in *Stim1 cKO* ameloblasts resulted in dysregulated expression of *Per2* that disrupts the TTFLs leading to abnormal circadian patterns including an upregulated *Bmal1* expression that maybe involved in the development of SOCE mediated AI. iCa^2+^, intracellular calcium; TTFL, transcription translation feedback loop; PKA, protein Kinase A; PKC, protein Kinase C; CREB, cAMP response element-binding protein; STIM1, Stromal interaction molecule 1; P, Phosphate group. This figure was created with images adapted from Servier Medical Art by Servier. Original images are licensed under a Creative Commons Attribution 3.0 Unported License.

Calcium signaling is particularly important in regulating the circadian expression of *Per1&2* genes as their promoters contain functional CREs (cAMP/Ca^2+^-response elements) (Shim et al., 2007). *In vivo* and *in vitro* studies of the SCN showed that calcium influx induces the activity of Ca^2+^/calmodulin-dependent protein kinase II (CaMKII), resulting in the activation of protein kinase A (PKA), and protein kinase C (PKC). These kinases then phosphorylate CREB in a circadian manner (Agostino et al., 2004; Hegazi et al., 2019). The phosphorylated CREB translocates into the nucleus where it recognizes the CRE of the Per genes promoters and induces their expression (Lee et al., 2010). Furthermore, it has been shown that SOCE is essential for activating CREB in multiple tissues including smooth muscles and immune cells (Avila-Medina et al., 2018; Martin-Romero et al., 2018). The fact that SOCE-deficient ameloblast showed significantly lowered levels of the *Per*2 genes suggests that control of CREB activity may represent one of the main mechanisms by which SOCE can directly affect the circadian TTFLs in ameloblasts, similar to the SCN and other cells (Figure 4). Indeed, other researchers have also postulated that SOCE may be one of the intracellular modulators that links calcium to the circadian clock both centrally and peripherally (Noguchi et al., 2012, 2017; O’Neill and Reddy, 2012). Additional studies are needed in ameloblasts to evaluate why SOCE disruption affected the *Per2* gene much more than *Per1*. Nevertheless, we postulate that *Per2* downregulation, which results in *Bmal1* upregulation, is one of the main molecular mechanisms involved in the *Stim1 cKO*-caused AI.

Another key finding from our work is the observation that *Stim1* cKO ameloblasts showed higher level of *Fbxl3* expression, a key component of the circadian clock. FBXL3 protein plays an important role in the negative feedback loop of the mammalian molecular circadian rhythm. In the nucleus, the FBXL3 protein targets the CRY2 proteins by ubiquitination to prevent them from inactivating BMAL1 (Huber et al., 2016). Moreover, FBXL3 plays a vital role in promoting Bmal1 transcription by inactivating the Bmal1 suppressor, REV-ERB alpha (Shi et al., 2013). Thus, upregulated levels of *Fbxl3* expression might also be one of the ways that lead to the over-expression of Bmal1 in *Stim1* cKO ameloblast.

The use of PCR arrays to investigate the genes that are differentially expressed in SOCE-deficient ameloblasts has brought forward genes that are also known to play a role in ameloblast differentiation and tooth morphogenesis, including *Tgf*-β*1* and *Mapk14*. TGF-β1 is a multi-functional growth factor with important roles in several biological processes, such as cellular proliferation and embryonic development, in addition to its role in the circadian clock signaling (Xu et al., 2018). Our RNA array and qRT-PCR analyses revealed that *Tgf-*β*1* is significantly downregulated in SOCE-deficient ameloblasts. Several previous studies had showed that SOCE plays an important role in regulating the TGF pathway activity in healthy and cancerous tissues (Chaudhari et al., 2017; Bhattacharya et al., 2018). In the context of amelogenesis, TGF-β1 is known to play a key role in the normal enamel development, and similar to STIM1 it appears to play an important role in regulating ameloblast function during enamel maturation through its interactions with Klk4 and matrix metalloprotease 20 (Kobayashi-Kinoshita et al., 2016). In fact, Cho et al. (2013) has shown that ameloblast specific deletion of its receptors has resulted in hypomature AI enamel phenotype with decreased mineral content concomitant with increased attrition and thinner enamel crystallites. This phenotype is not unsimilar to the enamel phenotype observed in other *Stim1* cKO mice (Eckstein et al., 2017; Furukawa et al., 2017; Said et al., 2019), suggesting that TGF-β1 dysregulation might be partly involved in the development of *Stim1* deletion-caused AI. On the other hand, *Mapk14* expression was significantly upregulated in *Stim1* deficient ameloblasts. MAPK14 has been shown to regulate SOCE both indirectly (through TGF-β1 and NF-kappa) or directly by phosphorylating *Stim1* (Martin-Romero et al., 2018). In ameloblasts, MAPK14 has been shown to play a role in regulating early tooth morphogenesis and its ectodermal tissue deletion results in abnormally shaped dental cusps and a profoundly hypoplastic enamel layer (Greenblatt et al., 2015).

Additionally, the present RNA profile studies revealed that SOCE disruption altered the expression of other circadian transcription factors with currently unknown functions in amelogenesis. These include: transcription Factor AP-2 Alpha (TFA2PA), nuclear receptor subfamily 2 group F member 6 (NR2F6), and early growth response proteins 1&3 (EGR1, EGR3). TFA2PA is expressed in ectoderm and migrating neural crest cell lineages, and plays an important role in early craniofacial morphogenesis (Mitchell et al., 1991). Liu et al. (2015) showed that TFA2PA is highly expressed during the presecretory stage of amelogenesis, and suggested that it participates in the differentiation of pre-ameloblasts to secretory ameloblast. Similar to STIM1, both EGR1, and NR2F6 were shown to be highly expressed during the maturation stage, yet their exact roles in amelogenesis are poorly understood (Tsuchiya et al., 2009; Lacruz et al., 2011). Moreover, EGR1 is a major regulator of STIM1 and its expression is induced by TGF-β1 in the maturational ameloblasts which may explain why it is downregulated in *Stim1* cKO (Tsuchiya et al., 2009; Samakai et al., 2016). These preliminary findings and related hypotheses need further confirmation and will be part of future studies. Nevertheless, we can conclude that the apparent differential stage-specific regulation of circadian clock related genes (downregulation of *Tgf*-β*1*, *Egr1*, and *Nr2f6* which are expressed in maturation stage, and upregulation of *Mapk14* and *Tfa2pa* which are expressed in pre-secretory and secretory stage) in *Stim1* cKO teeth supports the idea of complex downstream effects of altered SOCE signaling in ameloblasts. Our data suggests that SOCE phenotypic and genotypic effects throughout amelogenesis may be produced by directly modulating downstream molecular signaling pathways and by contributing to the circadian clock system dysregulation observed in this model.

The essential role of calcium signaling in regulating the circadian clock has been demonstrated in several studies which have reported that changes in the intracellular [Ca^2+^] not only contribute directly to the timekeeping mechanism of the central and peripheral clocks, but also that the intracellular [Ca^2+^] is regulated by the circadian clock suggesting complex regulatory feedback mechanisms between the two systems (Honma and Honma, 2003; Lundkvist et al., 2005; Enoki et al., 2017; Martí Ruiz et al., 2018). Consistently, two studies by Noguchi et al. (2012, 2017) demonstrated how autonomous circadian oscillations in i[Ca^2+^] correlated with the autonomous rhythmicity of *PER2* expression in murine primary fibroblasts and the SCN neurons, and that both *PER2* and intracellular[Ca^2+^] rhythms were abolished in SCN cells deficient in Bmal1. In addition, Chen et al. (2016) reported that CLOCK-BMAL1 heterodimers regulate the expressions and functions of the cardiac L-type calcium channel which play an important role in the cardiac electrogenesis and arrhythmogenesis. Moreover, it was found that Ubiquitin-Specific Protease 2 (Usp2), which is a clock output effector, is involved in regulating bodily Ca^2+^ homeostasis *via* controlling the expression of the intestinal Ca^2+^ channels (Pouly et al., 2016). All the above suggests that an inverse regulation (i.e., the circadian clock regulates calcium homeostasis and rhythmicity) may also exist. However, most of the studies examining this correlation have been conducted in the SCN. The crosstalk between calcium signaling and circadian pathways in the peripheral clocks remain greatly understudied. Our report further contributes in understanding the chronobiology of circadian peripheral clocks and the links to intracellular [Ca^2+^] signaling. More studies are needed to examine the role of circadian clock in directing Ca^2+^ rhythmicity and signaling in ameloblasts.

In conclusion, in this work we analyzed the circadian gene profiles in the ameloblasts collected from a SOCE-deficient teeth. We uncovered how SOCE disruption resulted in the dysregulation of multiple circadian rhythm genes which may have a direct impact on certain aspects of *STIM1* AI. Our study provides further evidence that ameloblast activity is tightly controlled by several molecular and circadian pathways, and highlights the need to investigate their interactions more thoroughly to achieve a better understanding of amelogenesis. Future studies including additional functional assays are needed to further examine the potential molecular crosslinks between calcium dynamics and the circadian pathways in ameloblasts.

## Data Availability Statement

All datasets presented in this study are included in the article/Supplementary Material.

## Ethics Statement

The animal study was reviewed and approved by University Animal Care Committee (UACC) of the University of Saskatchewan, Saskatoon, SK, Canada.

## Author Contributions

RS, SP, and PP conceived the study. RS performed the experimental procedures, data analysis, experimental designing, and manuscript writing. LL helped with the experimental procedures and data analysis, and provided scientific support. SP and PP provided scientific support and contributed to the experimental design, data analysis, and manuscript writing. All authors read and approved the submitted version of the manuscript.

## Conflict of Interest

The authors declare that the research was conducted in the absence of any commercial or financial relationships that could be construed as a potential conflict of interest.
