# Supplementary material for: Calcium Sets the Clock in Ameloblasts
Source: Front Physiol. 2020 Jul 31;11:920. doi: 10.3389/fphys.2020.00920 (PMC7411184; doi:10.3389/fphys.2020.00920)
Supplement: Supplementary file 1 [file Table_1.DOCX]

**Supplemental Table 1**

| \| **Primer** \|  \| \| --- \| --- \| | **Forward sequence** | **Reverse sequence** |
| --- | --- | --- | --- | --- |
| iCre | CTCTGACAGATGCCAGGACA | TCTCTGCCCAGAGTCATCCT |
| Stim1 Flox | CGA TGG TCT CAC GGT CTC TA | GCT CTG CTG ACC TGG AAC TA |

Specific mouse Primers designed for Genotyping

Specific mouse Primers designed for qRT-PCR

| \| **Primer** \|  \| \| --- \| --- \| | **Forward sequence** | **Reverse sequence** |
| --- | --- | --- | --- | --- |
| Clock | CAAAATGTCACGAGCACTTAATGC | ATATCCACTGCTGGCCTTTGG |
| Bmal1 | CCAAGAAAGTATGGACACAGACAAA | GCATTCTTGATCCTTCCTTGGT |
| Cry1 | CGAGATGCAGCTATCAAGAAGC | TGTCCGCCATTGAGTTCTATGA |
| Cry2 | TGGAGCAGTCTGGACAGTCA | AGTAGGAACCTCCATCGGTTG |
| Rora | ACGCCCACCTACAACATCTC | TCACATATGGGTTCGGGTTT |
| Nr1d1 | CTTCCGTGACCTTTCTCAGCA | TGTGCGGCTCAGGAACATCAC |
| Per1 | CAGTACTTCTCTTTCTACATCCTGAGGACCG | CATTGCTATCACTGGAGGAGCCAGG |
| Per2 | AGAACTTGTTGCTCCTGCTT | GGAAGCTTGTAAGGGGTGGT |
| Stat5a | CACCAGCACGTTCATCATCG | CACTCATTGCGGGTGTTCTC |
| Mapk14 | CTCAGTGTGCAGTTCAACTC | CTTCTGCCAGTTCTAGCTTC |
| Tgfb1 | AAGGACCTGGGTTGGAAGTG | TGGTTGTAGAGGGCAAGGAC |
| Fbxl3 | GAGGAGATGCTCAGGCTAAG | CGTGGGTGTCTAGGTATGTG |
| Gapdh | AACAGCAACTCCCACTCTTC | TGGTCCAGGGTTTCTTACTC |
