# Supplementary material for: Calcium Sets the Clock in Ameloblasts
Source: Front Physiol. 2020 Jul 31;11:920. doi: 10.3389/fphys.2020.00920 (PMC7411184; doi:10.3389/fphys.2020.00920)
Supplement: Supplementary file 2 [file Table_2.DOCX]

**Supplemental Table 2**

Differentially expressed genes that were deemed significant (*P<0.05*). Fold changes are ratios of gene expression levels (*Stim1* cKO ameloblats/Control ameloblast).

| **Gene Symbol** | **Fold Regulation** | **p Value** |
| --- | --- | --- |
| Arntl | **3.82** | **0.022445** |
| Chrnb2 | **2.73** | **0.006360** |
| Egr3 | **9.76** | **0.030109** |
| Fbxl3 | **8.86** | **0.031773** |
| Mapk14 | **3.59** | **0.006068** |
| Nfil3 | **2.24** | **0.000886** |
| Rorc | **3.49** | **0.003267** |
| Tfap2a | **4.28** | **0.018819** |
| Egr1 | **-2.32** | **0.011202** |
| Nr2f6 | **-3.20** | **0.005080** |
| Per2 | **-2.66** | **0.046747** |
| Rora | **-3.52** | **0.004929** |
| Stat5a | **-3.67** | **0.003419** |
| Tgfb1 | **-5.19** | **0.002386** |
